# High percentage of microbial colonization of osteosynthesis material in clinically unremarkable patients

**DOI:** 10.1002/mbo3.658

**Published:** 2018-12-03

**Authors:** Ludwig Knabl, Bettina Kuppelwieser, Astrid Mayr, Wilfried Posch, Michaela Lackner, Débora Coraҫa‐Huber, Adrian Danita, Michael Blauth, Cornelia Lass‐Flörl, Dorothea Orth‐Höller

**Affiliations:** ^1^ Division of Hygiene and Medical Microbiology Medical University of Innsbruck Innsbruck Austria; ^2^ Department of Orthopedic Surgery Experimental Orthopedics Medical University of Innsbruck Innsbruck Austria; ^3^ Department for Trauma Surgery Medical University of Innsbruck Innsbruck Austria; ^4^ Present address: Adrian Danita Clinica Medicala Synexus Bucharest Romania

**Keywords:** bath sonication, biofilm, electron scanning microscopy, osteosynthesis implants

## Abstract

Stabilization of fractures with internal fixation devices is a common procedure and implant‐associated infections are a dreaded complication. The exact pathomechanism is not completely understood; however, microbial colonization of osteosynthesis material is considered a trigger for infection. This study aimed to determine the colonization rate of osteosynthesis implants in patients with no clinical or laboratory signs of infection, using two methods, conventional culture and polymerase chain reaction (PCR) of sonication fluid. Fifty‐seven patients aged between 18 and 79 years without signs of infection who underwent routine removal of osteosynthesis devices between March 2015 and May 2017 were included in this study. Osteosynthesis material was investigated by sonication followed by cultivation of the sonication fluid in blood culture bottles and PCR analysis, simultaneously. Additionally, electron scanning microscopy was performed in nine representative implants to evaluate biofilm production. Thirty‐two (56.1%) implants showed a positive result either by culture or PCR with coagulase‐negative staphylococci being the most commonly identified microorganism (68.1%). Furthermore, the detection rate of the culture (50.9%) was significantly higher compared to PCR (21.1%). The scanning electron microscopy imaging demonstrated biofilm‐like structures in four of six culture and/or PCR‐positive samples. This study is the first, to the best of our knowledge, to demonstrate bacterial colonization of osteosynthesis implants in healthy patients with no clinical or laboratory signs of infection. Colonization rate was unexpectedly high and conventional culture was superior to PCR in microbial detection. The common understanding that colonization is a trigger for infection underlines the need for strategies to prevent colonization of implant material like antibiotic‐loaded coating or intraoperative gel application.

## 1 INTRODUCTION

Fractures are commonly fixed with indwelling devices and implant‐associated infections are serious complications (Darouiche, 2004). The complex process of bacterial adhesion on implants, which represents a trigger for implant‐associated infections, is influenced by various factors like environment, adhesion potential and virulence factors of bacteria, material properties of the implant, and proteins in the serum or tissue (Katsikogianni & Missirlis, 2004; Ribeiro et al., 2012). Particularly, the chemical composition of the implant as well as its surface charge and roughness, the degree of hydrophobicity, and the appearance of specific proteins on the surface seem to influence the process of initial attachment (Ribeiro et al., 2012). Over time bacteria organize in so called biofilms, highly structured matrices of extracellular polymeric substances which are built by a single or a community of bacterial species and offer an increased protection against antibiotics and host immune responses (Arciola et al., 2012; Trampuz & Zimmerli, 2006). Biofilm‐related infections are an issue of major concern in orthopedic and trauma surgery as they represent the majority of surgical infections (Coraca‐Huber et al., 2012; Mauffrey et al., 2016).

Detection of microorganisms on implant material is difficult due to biofilm formation. The bath sonication technique is a well‐established technique for dislodgement of adherent bacteria from endoprosthesis and other implants (Tunney et al., 1998). It increases the sensitivity of pathogen detection significantly compared to direct culturing of implant material (Trampuz & Zimmerli, 2006). Inoculation of sonication fluid into blood culture bottles (Portillo et al., 2015) and the additional use of PCR from the sonication fluid further enhance pathogen detection (Esteban et al., 2012). In literature, there are several reports on the detection of pathogens from infected hardware (Levy & Fenollar, 2012; Trampuz & Zimmerli, 2006; Yano et al., 2014); however, to the best of our knowledge, there are no data on the microbial colonization of implants in noninfected patients.

Thus, the aims of this study were to determine the rate of microbial colonization of osteosynthesis implants removed from patients without clinical infection and the comparison of culture and PCR method in the identification of bacteria in colonized osteosynthesis implants.

## 2 MATERIAL AND METHODS

### 2.1 Study design

Fifty‐seven patients aged 18–79 years who underwent routine removal of osteosynthesis devices after long bone fractures with no clinical infection were included in this prospective cohort study from March 2015 to May 2017. The study was approved by the ethics committee of the Medical University of Innsbruck (Nr. 290/4.7) and all patients signed the informed consent documents.

Patients with no clinical or laboratory signs (including C‐reactive protein) of infection at time of hardware removal were defined as noninfected and thus included in the study. All patients received an antibiotic prophylaxis with a cephalosporin at the time of implant installation according to guidelines. Patients under 18 years or over 80 years; patients with previous infections or osteomyelitis at the site of surgery; clinical, radiological, or laboratory abnormalities which refer to implant‐associated infections, uncontrolled diabetes mellitus, immune depression (including HIV infection) or systemic use of corticosteroids or immunosuppressive treatment for organ transplantation were excluded.

The hardware was removed as usual, based on a variety of indications and according to national guidelines. Implants were placed in sterile closed containers and transported within 1 hr to the routine, certified microbiological laboratory for investigation.

### 2.2 Microbiological methods

For sonication, the removed implants in the sterile containers were covered with sterile NaCl‐solution and shaken for 30 s. The BactoSonic^®^ biofilm sonication bath (Bandelin, Berlin, Germany) was filled with deionized water, the containers were submerged in water and the ultrasonic energy was applied for 1 min. After sonication, the containers were shaken again for 30 s. After this process, the sonication fluid was aspired with a sterile 10‐ml syringe and tested for pathogens by culture and PCR.

An aerobic and anaerobic blood culture bottle were filled with 10 ml of the sonication fluid, each, and incubated in the fully automated microbial detection system BacT/ALERT^®^ 3D (BioMerieux, Marcy‐l’Étoile, France) for a maximum of 7 days. In case of a positive signal, 10 μl of the fluid was cultivated on each of the four culture medium plates [chocolate agar, blood agar, MacConkey agar, and CDC‐blood anaerobic agar (all Becton Dickinson, Heidelberg, Germany)] and the plates were incubated for 24 hr (aerobic) and 48 hr (anaerobic) at 37°C. In case there was no growth of microorganisms on the culture plate, the blood culture bottle was incubated again till the maximum of 7 days. Bacterial identification was done by the MALDI‐TOF (Bruker Daltonics, Bremen, Germany) using the direct smear method. A score above 1.7 was considered valid. In case the score was below this threshold, the mass‐spectrometric identification was repeated or identification was done by DNA sequencing as described elsewhere (Grif et al., 2012).

Simultaneously, real‐time PCR followed by sequence analysis of the sonication fluid was performed with the IVD, CE‐certified SepsiTest^®^‐UMD kit (Molzym GmbH, Bremen, Germany) according to the manufacturer's instructions. Briefly, pathogens were enriched from the sonication fluid after degradation of human DNA as well as free microbial DNA by DNAse treatment. The remaining microbial DNA was isolated and purified by column‐based extraction. The PCR assays for analysis of the DNA eluates are based on primer sequences that bind conserved regions of the 16S (V3/V4 region) and 18S (V8/V9 region) rRNA genes of bacteria and fungi, respectively. The real‐time PCR was performed according to the manufacturer's instructions.

To exclude possible contamination of buffers and reagents used in the kit, an extraction control consisting of an empty DNA isolation column, that is processed analogously to the clinical samples, was performed in each run.

PCR amplicons were cleaned with ExoSAP‐IT and for sequencing the BigDye XTerminator purification kit (Applied Biosystems, USA) was used. DNA amplicons were sequenced with a 3500 Genetic analyzer (Applied Biosystems). Sequences were BLAST compared using SepsiTest^™^ BLAST tool (http://www.sepsitest-blast.de/de/).

### 2.3 Scanning electron microscopy

To assess the presence of biofilm‐like structures, nine representative samples were also investigated by scanning electron microscopy. Three parts of removed plates or screws of each patient were prepared for the scanning electron microscopy. The remaining parts of the implants were used for microbiological investigations. For scanning electron microscopy, the implants were immersed in 2 ml of glutaraldehyde 2.5% for fixation. After fixating for 24 hr at 4°C, the implants were dehydrated with an ascending alcohol series (50%–70%–80%–99.9% ethanol). Each step lasted 5 min. After the last step, the implants were placed in an incubator for drying. The dried samples were glued on aluminum pins with Leit‐C (Plano GmbH, Wetzlar, Germany). The pins were sputtered with Au using an automatic sputter coater (Agar Scientific Ltd, Stansted, Great Britain) for 1 min and analyzed by scanning electron microscopy (SEM, JSM‐6010LV, JEOL GmbH, Freising, Germany).

### 2.4 Statistical analyses

The results were analyzed by the use of GraphPad Prism (version 5) software. Student's *t* test was performed to compare the paired means of the two measurement groups. *P* values of <.05 were considered significant.

## 3 RESULTS

### 3.1 Demographic data

The mean age of the 57 study patients was 47 (19–79) years, 34 patients were females (59.6%). The implants were in situ for a median time of 427 (50–1,998) days. In 37 of the 57 samples (64.9%), the time in situ was >1 year. The most common regions of fracture were the wrist/forearm (*n* = 23) and the ankle (*n* = 17).

### 3.2 Microbiological results

Thirty‐two of 57 implant samples were found to be culture‐ and/or PCR‐positive (56.1%) and coagulase‐negative staphylococci were the most frequently detected organisms (68.1%).

In 29 (50.9%) of these 57 samples, 35 microorganisms were detected by culture (in six patients, two different organisms were found) (Table 1). Microbial identification by MALDI‐TOF revealed a valid result above the threshold (>1.7) in all cases. Coagulase‐negative staphylococci (*n* = 29) were the most commonly identified organisms in culture. Other identified organisms were *Propionibacterium acnes* (*n* = 2), *Staphylococcus aureus* (*n* = 1), *Streptococcus parasanguinis* (*n* = 1), *Bacillus thuringiensis* (*n* = 1), and *Clostridium perfringens* (*n* = 1).

**Table 1:** Microorganisms isolated from the sonication fluid by culture and PCR

Culture	PCR	Count (*n*)
*Staphylococcus epidermidis*	No organism	5
*Staphylococcus capitis*	No organism	3
*Staphylococcus hominis*	No organism	3
*Staphylococcus xylosus*	No organism	1
*Staphylococcus pettenkofferi*	No organism	1
*Streptococcus parasanguinis*	No organism	1
*Clostridium perfringens*	No organism	1
*Staphylococcus epidermidis* *Staphylococcus lugdunensis*	No organism	1
*Staphylococcus epidermidis* *Bacillus thuringiensis*	No organism	1
*Staphylococcus aureus* *Staphylococcus hominis*	No organism	1
*Staphylococcus capitis* *Staphylococcus hominis*	No organism	1
*Staphylococcus hominis* *Propionibacterium acnes*	No organism	1
*Staphylococcus epidermidis* *Staphylococcus xylosus*	*Bifidobacterium subtile*	1
*Staphylococcus epidermidis*	*Staphylococcus epidermidis*	1
*Staphylococcus hominis*	*Staphylococcus epidermidis*	1
*Staphylococcus capitis*	*Staphylococcus saccharolyticus*	1
*Staphylococcus epidermidis*	*Bifidobacterium subtile*	2
*Staphylococcus hominis*	*Anaerococcus vaginalis*	1
*Staphylococcus epidermidis*	*Corynebacterium tuberculostaticum*	1
*Propionibacterium acnes*	*Propionibacterium acnes*	1
No organism	*Propionibacterium acnes*	1
No organism	*Enterococcus faecalis*	1
No organism	*Streptococcus oralis*	1

PCR analysis detected pathogens in 12 samples (21.1%). *Bifidobacterium subtile* was the most commonly identified organism, being found in three samples. Other identified organisms were *Staphylococcus epidermidis* (*n* = 2), *Propionibacterium acnes* (*n* = 2), *Staphylococcus saccharolyticus* (*n* = 1), *Anaerococcus vaginalis* (*n* = 1), *Enterococcus faecalis* (*n* = 1), *Streptococcus oralis* (*n* = 1), and *Corynebacterium tuberculostaticum* (*n* = 1). In nine (15.8%) of these samples, both methods were positive; out of these, two samples were concordant on species‐level, two on genus‐level, and the other five samples were completely discordant. Fungal pathogens were not detected.

While sterile implants showed a median time in situ of 399 days (58–1,380), microbial‐positive implants were in situ for a median time of 452.5 days (50–1,998). However, there was no significant association between microbial detection and the in situ time of the implant (*p* = .43).

### 3.3 Scanning electron microscopy results

**Figure 1:**
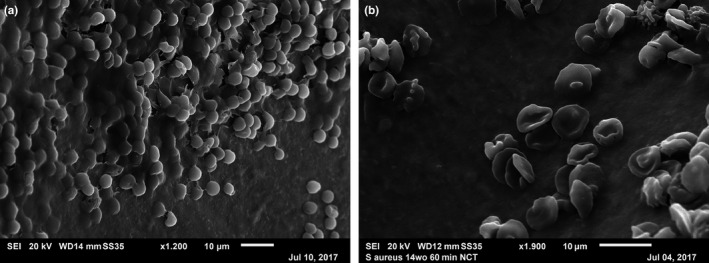
Representative scanning electron microscopy images of investigated samples. (a) shows biofilm‐like structure on the implant surface (x1.200); (b) shows an implant surface covered with blood cells (x1.900)

Nine of the 57 samples were also investigated by scanning electron microscopy. Biofilm‐like structures were found on the removed osteosynthesis material of four samples (Table 2) (Figure 1). In one case, culture and PCR were positive for coagulase‐negative staphylococci. In three of the four cases culture showed microbial growth with coagulase‐negative staphylococci (*n* = 2) or *Streptococcus parasanguinis* (*n* = 1), whereas PCR was negative. In three of the four biofilm‐positive cases, all three parts of the implants showed presence of biofilm‐like structures, whereas in one case, a biofilm was diagnosed in only one part of the implant (the residual two parts were biofilm negative). In two implant samples, no biofilm‐like structures were found although culture detected coagulase‐negative staphylococci. In the residual three implants no microorganisms were found by culture and/or PCR and as expected also scanning electron microscopy showed no biofilm‐like structures.

**Table 2:** Electron scanning microscopy results

Patient	Culture	PCR	Electron scanning microscopy		
			1^a^	2^a^	3^a^
1	*Staph. hominis*	No organism	Negative	Negative	Negative
2	No organism	No organism	Negative	Negative	Negative
3	No organism	No organism	Negative	Negative	Negative
4	No organism	No organism	Negative	Negative	Negative
5	*Staph. hominis*	No organism	Negative	Negative	Negative
6	*Staph. epidermidis*	No organism	Weak positive	Weak positive	Weak positive
7	*Strept. parasanguinis*	No organism	Positive	Positive	Positive
8	*Staph. capitis*	No organism	Negative	Negative	Positive
9	*Staph.capitis*	*Staph. saccharolyticus*	Positive	Positive	Positive

a Different parts (1, 2, 3) of the removed osteosynthesis implants.

## 4 DISCUSSION

To the best of our knowledge, this is the first study investigating presence of microorganisms on implants of noninfected patients, prospectively. Even more, the rate of microbial detection was much higher than expected with more than every second implant sample (56.1%) being positive in culture and/or PCR. Contamination as cause of these findings is very unlikely due to adherence to strict aseptic work flows during sampling and laboratory analysis. Additionally, randomly performed scanning electron microscopy examination of the removed implants showed biofilm‐like structures in four of six PCR or culture‐positive samples. Thus, we assume that the presence of microorganisms on the osteosynthesis material in this study represents colonization of the material. Patients with implant‐associated infections were excluded from the study.

In our study, coagulase‐negative staphylococci were the most commonly detected microorganisms (68.1%). Colonization of osteosynthesis material may pose a risk factor for development of implant‐associated infections. This assumption is supported by the fact that coagulase‐negative staphylococci are a common cause for implant‐associated infections (Ochsner et al., 2015). However, the onset of implant‐associated infections is a complex interplay between the host (including the osteosynthesis material) and the pathogen. Immune cells have only very limited access to the foreign body surface due to a lack of blood vessels on the implant (Zimmerli & Sendi, 2011). Furthermore, activity of immune cells is influenced by the simple presence of an implant. Zimmerli and colleagues demonstrated that the bactericidal and phagocytic capacity of polymorphonuclear leucocytes is significantly reduced in the presence of foreign bodies (Zimmerli et al., 1982). It is considered that interactions of granulocytes with the implant lead to a decrease in cell activity (Zimmerli et al., 1984). These conditions are very likely to favor colonization of implant material by bacteria.

Beside the host factors, the virulence factors of the colonizing pathogens are likely involved in the development of implant‐associated infections. Thus, it has been shown that mutations in the *agr* gene from *Staphylococcus epidermidis* leads to increased biofilm formation (Otto, 2009). Electron scanning microscopy has shown biofilm‐like structures in four of six culture and/or PCR‐positive implants underlining the colonization status and excluding the possibility of work‐flow contamination. However, in two culture‐positive implants, no biofilm‐like structures were detected. This finding could reflect a false negative microscopic result due to covering of the biofilm with blood or due to use of different samples for culture and electron microscopy. Whether presence of a biofilm determines the ability of microorganisms to cause infection was not assessed in this study.

Interestingly, culture detected microorganisms in 50.9% of implant samples, whereas PCR in 21.1% only. This result stands in conflict with various studies in which the additional use of PCR from the sonication fluid could increase the microbial detection rate compared with conventional culture methods only (Achermann et al., 2010; Esteban et al., 2012). However, these studies were performed in orthopedic implant infections and the reasons for the superiority of PCR were attributed to the ability of PCR to detect fastidious, noncultivatable or nonviable organisms, which might have been caused by a prior antibiotic therapy (Esteban et al., 2012; Gomez et al., 2012). The latter advantage of PCR is not evident due to investigation of noninfected and nonpretreated patients. However, another explanation for the low microbial detection rate of PCR might be the use of the small sample volume for the PCR assay (1 ml of sample for PCR vs. 10 ml for culture) especially when considering the fact that the microbial load in case of implant colonization is very likely to be lower than in case of an implant‐associated infection.

Beside the low detection rate of PCR in this study, the rate of discordant results within PCR and culture‐positive samples was high (77.8% discordance rate on species‐level, 55.6% on genus‐level).

In conclusion, this study is the first, to the best of our knowledge, to show the colonization status of osteosynthesis implants in a population of noninfected patients. The microbial burden on implant material was unexpectedly high with 56.1% showing presence of microorganisms by culture and/or PCR. In this study culture was superior in microbial detection compared to PCR. The finding of a high colonization rate in implants of noninfected patients is of importance when considering the common understanding that colonization is a trigger for infection. The results of our study may also give rise to protect implants from colonization by procedures like antibiotic‐loaded coating (Schmidmaier et al., 2006) or intraoperative gel application (Malizos et al., 2017).

## CONFLICT OF INTEREST

The authors declare no conflicts of interest.
